# Brain Calcifications: Genetic, Molecular, and Clinical Aspects

**DOI:** 10.3390/ijms24108995

**Published:** 2023-05-19

**Authors:** Edoardo Monfrini, Federica Arienti, Paola Rinchetti, Francesco Lotti, Giulietta M. Riboldi

**Affiliations:** 1 Dino Ferrari Center, Neuroscience Section, Department of Pathophysiology and Transplantation, University of Milan, 20122 Milan, Italy; 2 Foundation IRCCS Ca’ Granda Ospedale Maggiore Policlinico, Neurology Unit, 20122 Milan, Italy; 3 Columbia University Irving Medical Center, Center for Motor Neuron Biology and Diseases, Departments of Pathology & Cell Biology and Neurology, New York, NY 10032, USA; 4 The Marlene and Paolo Fresco Institute for Parkinson’s and Movement Disorders, Department of Neurology, NYU Langone Health, New York, NY 10017, USA

**Keywords:** primary familial brain calcification (PFBC), *SLC20A2*, *PDGFB*, *PDGFRB*, *XPR1*, *JAM2*, *MYORG*

## Abstract

Many conditions can present with accumulation of calcium in the brain and manifest with a variety of neurological symptoms. Brain calcifications can be primary (idiopathic or genetic) or secondary to various pathological conditions (e.g., calcium–phosphate metabolism derangement, autoimmune disorders and infections, among others). A set of causative genes associated with primary familial brain calcification (PFBC) has now been identified, and include genes such as *SLC20A2*, *PDGFB*, *PDGFRB*, *XPR1*, *MYORG*, and *JAM2*. However, many more genes are known to be linked with complex syndromes characterized by brain calcifications and additional neurologic and systemic manifestations. Of note, many of these genes encode for proteins involved in cerebrovascular and blood–brain barrier functions, which both represent key anatomical structures related to these pathological phenomena. As a growing number of genes associated with brain calcifications is identified, pathways involved in these conditions are beginning to be understood. Our comprehensive review of the genetic, molecular, and clinical aspects of brain calcifications offers a framework for clinicians and researchers in the field.

## 1. Introduction

Brain calcifications (BC) are intracranial calcium deposits localized in the brain parenchyma and its microvasculature [1,2]. Their prevalence ranges from 1% in young individuals up to 38% in elderly subjects [2,3,4]. Calcified areas are easily identified by clinicians as hyperdense alterations on brain CT. A certain degree of intracranial calcifications, particularly of the basal ganglia, pineal gland, choroid plexus, and habenula, can be considered a normal phenomenon associated with aging [2]. Indeed, BC are often incidental findings on neuroimaging of asymptomatic individuals; however, they can also be associated with many genetic and acquired disorders [5,6].

BC can be primary, as observed in several early- and late-onset genetic syndromes, or can be secondary to systemic alterations of phosphate–calcium metabolism (genetic and also acquired forms), intrauterine (e.g., TORCH) and post-natal infections (e.g., neurocysticercosis), hypoxic-ischemic injuries, toxic exposures (e.g., lead), brain tumors (e.g., oligodendrogliomas), and autoimmune disorders (e.g., systemic lupus erythematosus) [2,5].

Although large-scale epidemiological studies are lacking, the most common neurological disorder associated with late-onset BC is traditionally known as Fahr disease [5]. It is clinically defined by the variable presence of movement disorders, recurrent headaches, and psychiatric manifestations, in association with the presence of bilateral BC, most commonly in the basal ganglia, but also in the subcortical white matter, thalamus, and cerebellum [1]. Historically, different names have been used to refer to this neurological condition, including: idiopathic basal ganglia calcification (IBCG), bilateral striopallidodentate calcinosis (BSPDC), and primary familial brain calcification (PFBC) [1]. PFBC is currently the most commonly used term and defines a genetically confirmed neurodegenerative disorder with BC, in absence of a secondary cause. The term “primary bilateral brain calcification” has been proposed to define both inherited and sporadic cases, facilitating the differentiation between primary and secondary forms, and including all possible anatomical distributions of calcium deposits [7].

Many other heterogeneous early-onset complex genetic syndromes manifest BC as a part of their clinical presentation, almost invariably in association with other signs and symptoms, including neurodevelopmental delay, intellectual disability, epilepsy, dystonia, dysmorphisms, and varied systemic involvement [1,5].

No disease-modifying therapies are available for primary brain calcifications, nor have definite pharmacological targets been identified. Therefore, it is important to increase awareness of the clinical and molecular aspects of this group of disorders in order to facilitate diagnosis and stimulate translational research in this often overlooked field. In this review, we describe the genetic, molecular, and clinical aspects of primary BC, with the main focus directed on a detailed characterization of PFBC. In addition, an overview of the early-onset genetic syndromes associated with BC is provided.

## 2. Genetics of Brain Calcification

In the past decades, genetic etiology of IBGC was suspected based on the identification of several familial clusters [8,9,10,11]. However, no genetic cause of IBGC was recognized before the identification in 2012 of *SLC20A2* as the first associated gene [12]. Since then, six genes have been definitively linked with PFBC: four inherited in an autosomal dominant manner (*SLC20A2*, *PDGFB*, *PDGFRB*, and *XPR1*) and two displaying an autosomal recessive inheritance (*MYORG* and *JAM2*) [13,14,15,16,17]. Very recently, a novel gene (i.e., *CMPK2*) has been proposed to be associated with autosomal recessive brain calcifications and this awaits confirmation in independent studies [18]. A review of data from 555 genetically diagnosed patients with PFBC revealed the following frequency of mutations: *SLC20A2*~60%, *MYORG*~13%, *PDGFB*~13%, *PDGFRB*~6%, *XPR1*~6%, *JAM2*~2% [19,20]. A brief genetic characterization of each these genes is provided below in association with the genetic evidence deriving from knock-out or hypomorphic animal models.

*SLC20A2* (chr. 8p11.21, 11 exons) encodes the sodium-dependent phosphate transporter 2 (652 amino acids), which plays a fundamental role in cellular phosphate transport. Several pathogenic variants have been reported, including missense, nonsense, splice-disruptive, small indels, and gross deletions. Pathogenic variants have been shown to impair uptake of phosphate (loss-of-function mechanism of disease) [12]. Interestingly, a complete Slc20a2 knockout mouse showed extensive, bilateral calcifications in the thalamus, basal ganglia, and cortex, recapitulating the human disease [21].

*MYORG* (chr. 9p13.3, one coding exon) encodes the myogenesis regulating glycosidase (putative) (714 amino acids), a protein located in the endoplasmic reticulum and nuclear membranes whose functions remain poorly defined, but are probably linked to protein glycosylation [22]. Many pathogenic variants have been described, including missense, nonsense, and small indels. The presence of biallelic nonsense mutations of *MYORG* in PFBC patients strongly suggests that a functional loss of *MYORG* is associated with brain calcifications. Indeed, Myorg-KO mice and zebrafish develop progressive brain calcifications [14,23].

*PDGFRB* (chr. 5q32, 23 exons) encodes the platelet-derived growth factor receptor beta (PDGFR-β) (1106 amino acids), a cell surface tyrosine kinase receptor for mesenchymal cell mitogens. Several pathogenic variants are known, including missense and start-loss mutations. Pdgfrb-deficient mice lack pericytes, essential for blood–brain barrier (BBB) formation, and display increased vascular permeability [24].

*PDGFB* (chr. 22q13.1, six exons) encodes the platelet-derived growth factor subunit B (241 amino acids), which is a potent mitogen for cells of mesenchymal origin, by binding and activating PDGF receptor tyrosine kinases such as PDGFR-β. A number of pathogenic variants have been reported in association with PFBC, including missense, nonsense, splice-disruptive, start loss, stop loss, and gross deletions. Hypomorphic Pdgfb mice develop brain calcifications that correlate with the degree of pericyte and BBB deficiency [17].

*XPR1* (chr. 1q25.3, 15 exons) encodes the xenotropic and polytropic retrovirus receptor 1 (696 amino acids), which plays a role in phosphate homeostasis and mediates phosphate export from the cells. Few pathogenic variants have been reported, which include missense and splice disruptive. Remarkably, the majority of these are located in the SPX putative regulatory domain of the protein [15,25].

*JAM2* (chr. 21q21.3, 10 exons) encodes the junctional adhesion molecule 2 (298 amino acids), which is localized at the tight junctions of both epithelial and endothelial cells and mediates heterotypic cell–cell interactions [26]. Being the most recently described gene, few pathogenic variants have been reported, including missense, start-loss, nonsense, and splice-disruptive. Pathogenic variants lead to reduction/absence of *JAM2* protein, consistent with a loss-of-function mechanism. The brain calcification phenotype is replicated in the Jam2 complete knockout mouse [13].

### Genetics of Early-Onset Syndromes Associated with Brain Calcifications

Calcifications in the basal ganglia and other brain structures are also observed in several genetic diseases with normal calcium–phosphate metabolism. This clinically and genetically highly heterogeneous group of syndromes includes mitochondrial diseases (predominantly mtDNA mutations), Aicardi–Goutières syndrome (*ADAR*, *RNASEH2A*, *RNASEH2B*, *RNASEH2C*, *SAMHD1*, *TREX1*, *IFIH1*), Coats plus syndrome (*CTC1*), leukoencephalopathy with calcifications and cysts (*SNORD118*), Cockayne syndrome (*DDB2*, *ERCC1*, *ERCC2*, *ERCC3*, *ERCC4*, *ERCC5*, *ERCC6*, *ERCC8*, *GTF2H5*, *MPLKIP*, *POLH*, *XPA*, *XPC*) and many others. Deep clinical phenotyping is crucial before genetic testing in this context [27]. In the absence of specific diagnostic clues for a particular form (see below) the best genetic approach is next-generation sequencing (e.g., gene panels or exome sequencing) [27]. A detailed genetic description of every one of the many genes involved in these syndromes is beyond the scope of this review. A complete list is provided in Table 1.

## 3. Molecular Mechanisms of Brain Calcifications

The pathophysiology of PFBC is profoundly linked to the loss of integrity of the BBB and should be distinguished from the calcification processes that take place in secondary brain calcifications, in which calcium deposition develops via two main pathways: dystrophic calcification resulting from membrane disruption and uncontrolled calcium entry (hypoxic ischemic injury, intrauterine infections), and calcium–phosphate metabolism alterations (pseudohypoparathyroidism and pseudopseudohypoparathyroidism, mainly linked to loss of function mutations of GNAS, involved in the intracellular transmittal pathway of PTH) [28].

**Figure 1:**
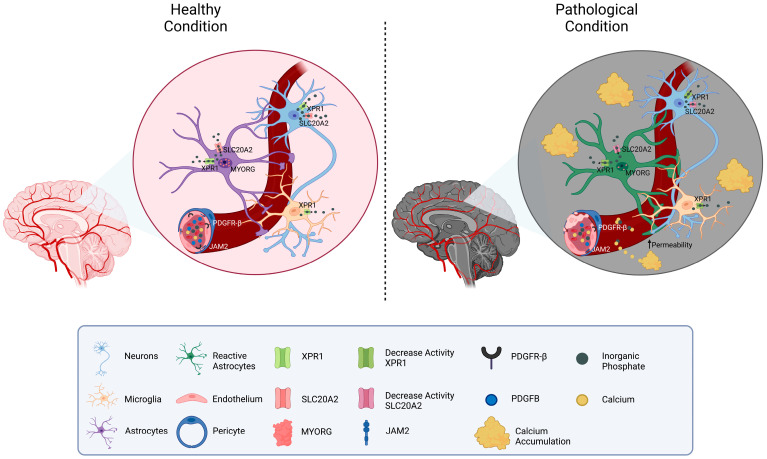
**Schematic representation of the molecular mechanisms related to PFBC-genes**. A neurovascular unit is depicted. The localization of the genes related to PFBC (*SLC20A2*, *PDGFB*, *PDGFRB*, *XPR1*, *MYORG*, and *JAM2*) is shown in the figure. On the left side, physiological Pi homeostatic processes are illustrated (mostly modulated by SLC20A2 (import) and XPR1 (export)). On the right side, pathological conditions due to gene mutations’ protein down-regulation are depicted. Reduced levels of MYORG result in impaired function and confirmation of astrocytes. Disfunction of PDGFB, PDGFRB, and JAM2 causes increased permeability of the vessels and calcium leakage. Interstitial accumulation of Pi and calcium is responsible for the formation of brain calcifications.

Considering the functions of the known causative genes, the pathology of PFBC can be ascribed to altered phosphate transportation or impaired permeability through the BBB, which both result in increased inorganic phosphate (Pi) levels in CSF and brain interstitial fluid. The excess of Pi induces its deposition in the form of calcium hydroxyapatite (Ca_10_[PO_4_]6[OH]_2_) in the vascular extracellular matrix (Figure 1).

The reasons for the greater basal ganglia vulnerability to calcium deposition are not completely understood but may depend on the higher expression of the causative genes in the basal ganglia neurovascular system, as well as on the peculiar vascularization of these areas [29].

*SLC20A2* and *XPR1* operate directly in inorganic phosphate transportation: the former, mainly expressed by neurons, astrocytes, and vascular smooth muscle cells (VSMC), imports extracellular Pi into the cell mediating its transportation from CSF to blood; the latter exports intracellular Pi out of cells such as neurons, astrocytes, and microglia [15]. Mutations of these transporters lead to the accumulation of extracellular Pi, due to reduced Pi internalization in *SLC20A2* mutants, and to intracellular accumulation with subsequent down-regulation of uptake in *XPR1* mutants. It has been hypothesized that persistent elevation in extracellular Pi may induce intracranial calcification, possibly via trans-differentiation of vascular smooth muscle cells (VSMCs) into osteoblast-like cells [30].

Conversely, *PDGFRB*, *PDGFB*, *MYORG*, and *JAM2* operate in the brain neurovascular unit (made of neurons, astrocytes, endothelial cells, pericytes, and VSMCs) and indirectly impair the BBB permeability [31]. PDGFR-β is expressed mainly by pericytes and VSMCs, while PDGFB is predominantly released by the endothelium, enabling the recruitment of PDGFR-β-expressing cells and thus promoting the wrapping of the cerebral vessel [32]. Deficiency of PDGF-B/PDGFR-β signaling causes alteration in pericyte recruitment and endothelial cell morphology, leading to increased vascular permeability. MYORG is predominantly expressed in astrocytes and localized in the endoplasmic reticulum; though presumed to act as an α-glucosidase, its molecular function remains largely unknown [22]. Astrocytic endfeet connect the endothelial cells to the extracellular matrix and neurons, thus exerting an important function in the maintenance of the BBB. Speculatively, intracellular ER alterations may impair astrocytes’ properties and conformation, causing BBB disruption. JAM2 is a key component of the tight junctions between endothelial cells in the neuro-vascular unit. *JAM2* loss-of-function causes disarrangement of tight junctions, leading to high Pi leakage from the blood to the brain tissue [13].

Concerning genetic brain calcifications other than PFBC, at least three additional pathogenetic mechanisms haver been identified:

1. **Cerebral microangiopathies and gliosis**, resulting from the malfunctioning of cellular systems fundamental in vessels’ stability and/or angiogenesis, such as small nucleolar RNA involved in ribosome biogenesis in leukoencephalopathy with calcification and cysts (LCC) [33], telomeres in Coats plus syndrome, nucleases in Aicardi–Goutières syndrome and type IV collagen in *COL4A1*-related disease [33,34,35,36].
2. **Reaction to microgliopathy and neuronal loss**, often seen in more complex encephalopathies such as Cockayne syndrome where microscopic examination has highlighted the presence of both iron and calcium deposits, or the NRROS-related disorders, Adams–Oliver syndrome and Nasu–Hakola disease [37].
3. **Mitochondriopathies**, where brain calcification may be attributed to co-present endocrine alterations (hypoparathyroidism) and/or to progressive cell degeneration caused by mitochondrial dysfunction in the brain [38].

The underlying pathogenetic mechanisms of ectopic/dystrophic calcifications of non-cerebral soft tissues are extremely different and are mainly associated with systemic mineral imbalance (i.e., hyperparathyroidism) or tissue alteration and necrosis (i.e., myositis ossificans) [39].

## 4. Clinical Aspects

### 4.1. Primary Familial Brain Calcifications: Clinical Aspects

Significant overlap is found in the clinical presentation of patients with PFBC caused by mutations of the monogenic forms identified so far (including *SLC20A2*, *PDGF*, *PDGFRB*, *XPR1*, *MYORG*, *JAM2*). Characteristic features of PFBC include motor symptoms such as hypokinetic (i.e., Parkinsonism) more than hyperkinetic (chorea, dystonia, or ataxia) movement disorders, seizures, pyramidal signs, cognitive decline, and psychiatric symptoms (such as behavioral changes, depression, anxiety, ADHD, psychosis, or bipolar disorders) [19]. Dysarthria and dysphagia have also been reported [26,40,41,42,43]. Headache (migraine with and without aura) and dizziness are often present and may be the presenting features prompting imaging study and thus diagnosis in subjects who are otherwise asymptomatic [41,44].

According to a recent extensive review of all the reported cases of PFBC, headache is very frequent (about 30–40% of cases) in *PDGFB*, *SLC20A2*, and *PDGFRB* mutation carriers [19]. Cognitive deficits are found in up to 50% of subjects with biallelic *JAM2* variants, followed by *MYORG*, and in more than one-third of cases of *XPR1*, *PDGFB*, *SLC20A2*. Parkinsonism is present in up to 80% of subjects with *JAM2*, in about 30% of cases of *XPR1*, *SLC20A2*, *MYORG*, and less frequently in *PDGFB*, *PDGFRB* mutation carriers. Other movement disorders, including chorea and dystonia, display lower frequency (less than 15% of cases). Ataxia is recurrent in *JAM2* cases (in up to 60% of subjects) and MYORG, and less frequent in *PDGFB*, *XPR1*, or *SLC20A2*. Psychiatric manifestations mainly include depression, psychosis, or personality changes. Depression is more frequent and is found especially in *PDGFB*, *MYORG*, and *PDGFRB* carriers, and less in other forms. Headaches are very common (about 30–40% of cases) in *PDGFB*, *SLC20A2*, and *PDGFRB* carriers. Finally, dysarthria is a frequent finding in subjects with biallelic variants of *MYORG* (more than 70% of cases), as well as in *JAM2* and *XPR1* mutation carriers (Table 1) [19].

In carriers of pathogenic variants of *SLC20A2*, additional features have been reported including forms of tremor (head tremor, intention tremor of the upper limbs), blepharospasm, torticollis, facial palsy, apraxia, palilalia, myoclonus (described as mostly cortical), cramps, active denervation at electromyographic (EMG) recording, polyneuropathy, syncope, as well as ischemic episodes (both transitory and stroke) [40,41,44,45,46,47,48,49,50,51]. Seizures have been described as both grand mal generalized or focal. Interestingly, one subject was described presenting a classical form of paroxysmal kinesigenic choreoathetosis with response to carbamazepine, as classically seen in subjects with PRRT2-related paroxysmal kinesigenic dyskinesia (PKD) [42]. PKD with response to carbamazepine was also reported in one subject with mutations of the *PDGFRB* gene [52]. Carriers of pathogenic variants of this gene also had episodes of urinary incontinence, neuropathy, cramps, and restless leg syndrome [46,49,53]. In subjects with PDGFB mutations, spasmodic adductor dysphonia has been reported, migraine is usually not associated with aura, and chorea and Parkinsonism have so far been found with similar frequency [17,54,55,56]. Neuropathy has also been described in subjects with *JAM2* mutations [26].

In these subjects, Parkinsonism is often levodopa responsive, with subjects developing motor fluctuations and dyskinesia [16,41,43,50,57]. Freezing of gait has also been observed in subjects with *SLC20A2* mutations [41]. A phenotype resembling supranuclear gaze palsy (PSP) with poor response to levodopa was reported in one patient with *PDGFRB* mutation who presented symptoms at age 79 [58].

For the autosomal forms of PFBC, age of onset of clinical manifestation spans from childhood (first decade) to the 80s, with more frequent presentation between the fourth and sixth decades [19]. Among these forms, *PDGFB* seems to be related to an earlier age of onset (first or second decade), while XPR1 has been found to manifest only after the third decade [19]. Considering autosomal recessive forms of PFBC, age of onset of *JAM2*-manifesting carriers spanned between 8 and 38 years of age. *MYORG* has been reported in subjects with age of onset spanning from the first to the eighth decade [19]. Gender is equally represented in all the genetic forms of PFBC [19].

Two-thirds of carriers usually manifest symptoms, with higher penetrance for the recessive forms (*JAM2* and *MYORG*) and *PDGFB*, followed by *XPR1*, *SLC20A2*, and less than 50% for *PDGFRB* [19]. Intrafamilial variability can be observed for all these genes, both in terms of age of onset as well as clinical presentation, arguing against a strong genotype–phenotype correlation [40]. However, gene variants, clinical traits, and/or age of onset or both showed consistency among family members in certain families [16,44,48,50,56,59].

Interestingly, two identical female twins with mutations of the *PDGFRB* gene showed a similar clinical phenotype (characterized mostly by paresthesia, cramps, and congenital atrial septal defect) and only five-year difference in the age of onset (49 and 54 years) [59]. Both twins presented brain calcification in the white matter, but one presented more extensive calcification in the basal ganglia (with later age of onset) [59].

In PFBC, brain calcifications are reported in the subcortical white matter as well as in the basal ganglia and cerebellum. Interestingly, affected *PDGFB* carriers presented more severe calcification in the thalamus, cerebellum, and white matter, as well as calcifications outside the basal ganglia, compared with non-affected carriers [19]. In these forms, calcification is a progressive process, so that the extent of brain calcification has a tendency to increase with age [19]. Of note, about 75% of the heterozygous carriers of *MYORG* were found to present brain calcifications despite remaining clinically asymptomatic, suggesting a dosage effect related to this gene [60,61]. A genotype-to-phenotype correlation has been observed more in regards to the extent of brain calcifications then in terms of severity of clinical presentation [19]. Of note, pontine calcifications are typically overserved in subjects with mutations or *MYORG* [62]. In subjects with PFBC, serum levels of calcium, parathormone (PRH), and vitamin D were found to be within range [26,41,42,50,51,55,57,63,64,65,66,67].

Whether the brain calcifications are the cause of these conditions or an epiphenomenon is still not clear. Interestingly, in PFBC the extent of brain calcification does not necessarily correlate with the severity of clinical symptoms. Indeed, these forms present a reduced penetrance and extensive brain calcifications can be found in asymptomatic subjects.

The current knowledge on the contribution of additional genetic variants in adjunct to the classical PFBC genes in terms of disease penetrance and phenotypic presentation is very limited. Cases were reported of two sisters presenting with basal ganglia calcifications and generalized epilepsy as main phenotype carrying mutations in the *SLC20A2* and in the *CHRNB2* genes (known to be associated with AD frontal lobe epilepsy) and a young boy with basal ganglia calcification and refractory epilepsy carrying mutations in the *SLC20A2* and in the *SCN2A* genes [68,69]. In addition, a small deletion on chromosome 8 including the *SLC20A2* and *THAP1* genes (associated with dystonia type 6) was considered responsible for a predominant dystonic phenotype with earlier age of onset compared with carriers of *SLC20A2* alone [70,71,72,73].

Compared with classical forms of PFBC, a patient with a mutation in the *CARS* gene and a variant of uncertain significance in the *PDGFRB* gene showed reduced levels of calcium in the peripheral blood [74]. Mutations of the *CASR* gene are associated with hypoparathyroidism, hypocalcemia, and relative hypercalciuria consistent with the low levels of calcium observed in this subject [75].

Finally, one rare case of digenic mutation of both *SLC20A2* and *PDGFRB* genes presented with an early onset (5 years) and severe basal ganglia and frontal calcification, while the heterozygous parents showed only mild brain calcification and no symptoms [76]. A role for digenic variants in determining the penetrance of the disease and the phenotypic presentation has been suggested by the abovementioned studies and warrants further investigation.

### 4.2. Other Forms of Brain Calcifications: Clinical Aspects

Brain calcifications can be one of the features of (mostly) pediatric encephalopathy due to a number of different genes (summarized in Table 1). These are usually complex conditions that may present with developmental delay and complex neurological presentations, including combinations of pyramidal signs, hypo or hyperkinetic movement disorders, ataxia, epilepsy, and cognitive impairment (such as speech delay/absence). From a radiological perspective, calcification of the basal ganglia, cerebellum, and white matter calcifications, as well as leukoencephalopathy are usually present.

In certain instances, specific radiological characteristics are detrimental to guide the differential diagnosis. The presence of subcortical cysts is suggestive of a diagnosis of LCC due to compound heterozygous variants in the *SNORD118* gene, or of Coats plus syndrome (cerebroretinal microangiopathy) due to mutations in the conserved telomere maintenance component 1 (*CTC1*) gene [77,78]. In Coats plus syndrome, LCC is associated with systemic microvascular manifestation, such as cutaneous or retinal telangiectasia, as well as vascular abnormalities causing gastrointestinal, hepatic, and bone marrow hemorrhage, and osteosclerotic lesions [78]. Cystic degeneration of the white matter, associated with calcification and leukodystrophy, was found in autoptic brains of patients with the NRROS-mutation associated syndrome, Rajab interstitial lung disease with brain calcifications-2, and *RNASET2*-related leukoencephalopathy [79,80,81].

Another difference between PFBC and other encephalopathies with brain calcification is the natural history of these conditions. While PFBCs usually manifest after the second or third decade, or earlier in life but after a normal development, other encephalopathies with brain calcification can present at birth with progressive developmental delay, such as in Cockayne syndrome [82]. Some forms present a biphasic progression (such as Aicardi–Goutières syndrome) while others are static encephalopathies (such as Adams–Oliver syndrome 2) [77,83].

Certain mitochondrial conditions, including the myoclonus epilepsy with ragged-red fibers (MERRF), Kearns–Sayre disease, and Leigh syndrome, can present brain calcifications [84,85,86,87,88,89]. Of note, since these conditions are caused by mutations in the mitochondrial DNA, patients also present classic mitochondrial features (such as short stature, optic atrophy, hearing loss, ptosis, diabetes mellitus, cardiomyopathy, neuropathy, myopathy, as well as lactic acidosis) and a matrilinear heritability that can help in the differential diagnosis [84,85,86,87,88,89].

Characteristic patterns of calcifications are found in patients harboring *CLDN5* mutations who present involvement of the vertical pontine white matter bundles, other than cortical and basal ganglia calcifications [90].

Other conditions associated with brain calcifications can present in association with cutaneous abnormalities, as well as dysmorphism, often involving the extremities, and specific inflammatory signatures. Onset of neurological symptoms after initial normal developmental, post-natal microcephaly, sterile pyrexia, chilblain lesions, and characteristic interferon signature in peripheral blood are suggestive of one of the forms of Aicardi–Goutières syndrome [91]. Persistent pancytopenia and dyskeratosis are consistent with mutation of genes involved in controlling telomere length and causing Hoyeraal Hreidarsson syndrome and family of diseases called dyskeratosis congenita that can present with severe childhood onset of progressive neurological deterioration and brain calcifications [92]. Erythematous nodular-like lesions, lipodystrophy, extremity joint deformity, specific inflammatory signature, and hyperpyrexia are found in Nakajo Nishimura syndrome, so far described only in Japanese patients [93]. Frequent association with autoimmune conditions is found in spondyloenchondrodysplasia with immune dysregulation syndrome, while frequent infection and susceptibility to TBC is found in immunodeficiency 38 with basal ganglia calcification syndrome [94,95]. Interstitial lung symptoms are, instead, characteristic of Rajab interstitial lung disease with brain calcifications 1 and 2 [80,96].

Cartilage abnormalities with dysmorphism, such as facial dysmorphism and brachytelephalangism are characteristics of Keutel Syndrome [97]. Dysmorphic features and terminal transverse limb defects can also be found in Adams–Oliver syndrome 2 where brain MRI shows characteristic periventricular calcifications and ventricular enlargement [83,98]. Hydrocephalus and basal ganglia calcifications are also found in male subjects with Fried syndrome [99,100,101].

Some conditions present a more catastrophic course, as in the case of the *JAM3*-related condition where the vascular brain disease that causes cerebral calcifications is also responsible for lethal hemorrhages [102]. Prominent early onset spastic paraplegia with brain calcifications, white matter abnormalities, and corpus callosum abnormalities are present in spastic paraplegia 56, with characteristic pseudoxanthoma elasticum due to calcium accumulation in the tendons (usually in the neck) [103].

Early onset Parkinsonism and intellectual disability with mild calcifications of the basal ganglia can be found in patients with sporadic or hereditary (X-linked) juvenile Parkinsonism due to mutations of the *RAB39B* gene [104,105]. Adult-onset neurodegeneration with early onset dementia and Parkinsonism is also found in Nasu–Hakola disease where brain atrophy and white matter abnormalities are associated with basal ganglia calcifications and cystic lesions of the bones [106].

Interestingly, genetic mutations have been identified in patients that resemble either congenital infection (pseudo-TORCH 1 and 2) or pseudohypoparathyroidism [107]. Finally, in some cases, brain calcification is due to genetic metabolic conditions that can benefit from prompt recognition and treatment, such as in inborn error of folate metabolism or 3-hydroxyisobutyric aciduria, for alleviating or preventing some of the symptoms [108,109]. Adult brain calcification findings presenting with vascular events (ischemic or hemorrhagic) or migraine are also characteristic of the spectrum of disorders associated with collagen *COL4A1* and *2* mutations [110,111,112].

## 5. Conclusions

Brain calcification can represent the epiphenomenon of a large number of systemic and genetic conditions. Associated clinical presentation (such as dysmorphism, cutaneous abnormalities, or immunological traits) can help in the process of differential diagnosis (Table 2). Systemic conditions, such as impaired calcium metabolism, infectious, and autoimmune/inflammatory conditions should always be considered in the diagnostic process, as they are curable.

There is no strong correlation between the genetic mutations and specific patterns of calcium deposition in the brain. However, additional findings such as brain cysts or prominent white matter abnormalities can be suggestive of specific forms.

Interestingly, many of the genes responsible for these forms are involved in mechanisms of angiogenesis and in the BBB, suggesting common pathways that could be tackled by therapeutic approaches to these conditions. A deepening in the understanding of the genetic architecture and the identification of new genetic forms of brain calcification will be crucial in this direction.

The most common genetic forms of brain calcifications (PFBC) can present with very mild and non-specific symptoms (such as headache or vertigo) and this diagnosis is likely to be overlooked in a large number of subjects. A role for digenic variants in determining the penetrance of the disease and the phenotypic presentation has been suggested by a few works in the literature and it warrants further investigation.

## Figures and Tables

**Table 1:** **Genetic syndromes associated with brain calcifications**—The table summarizes the genetic forms presenting brain calcifications. Demographic, clinical, and imaging traits for each syndrome are summarized in the table. MOI = mode of inheritance, AD = autosomal dominant, AR = autosomal recessive, mt = mitochondrial, XLR = X-linked recessive, IC = isolated cases. For the specific references of each condition, please refer to the main text.

Gene (Age of Onset (Range, Years)/Penetrance (%)/MOI)	Main Neurological Symptoms	Other Symptoms	Brain Calcification
***SLC20A2*** (1–84/60%/AD)	>20%: Parkinsonism, cognitive deficit, headache 10–20%: Tremor, dysarthria, dystonia <10%: Depression, psychosis, ataxia, anxiety, seizure, chorea		Basal ganglia, subcortical white matter, and dentate nuclei
***PDGFB*** (1–70/86%/AD)	>20%: Headache, cognitive deficit, depression 10–20%: Parkinsonism, psychosis, ataxia, tremor, chorea, anxiety <10%: Dystonia, dysarthria, seizure		Basal ganglia, subcortical white matter, and dentate nuclei
***PDGFBR*** (6–82/48%/AD)	>20%: Headache, cognitive deficit 10–20%: Depression, Parkinsonism <10%: Tremor, anxiety, dysarthria, seizure, psychosis, ataxia, tremor, chorea, dystonia		Basal ganglia, subcortical white matter, and dentate nuclei
***XPR1*** (16–79/70%/AD)	>20%: Cognitive deficit, Parkinsonism, dysarthria 10–20%: Tremor, ataxia <10%: Headache, anxiety, seizure, psychosis, depression, dystonia, chorea		Basal ganglia, subcortical white matter, and dentate nuclei
***MYORG*** (8–87/91%/AR)	>20%: Dysarthria, cognitive deficit, ataxia, parkinsonism, psychosis, tremor 10–20%: Depression <10%: Headache, dystonia, chorea, seizure, anxiety		Basal ganglia, subcortical white matter, and dentate nuclei
***JAM2*** (7–50/85%/AR)	>20%: Parkinsonism, ataxia, cognitive deficit, dysarthria, seizure <10%: Dystonia		Basal ganglia, subcortical white matter, and dentate nuclei, midbrain
**Mitochondrial conditions** (MERRF, Leigh syndrome, KSS—mtDNA) (Childhood, adulthood/full/matrilinear)	Epilepsy, myoclonus, ataxia, spasticity Dysphagia (LS)	Short stature, optic atrophy, hearing loss, ptosis, diabetes mellitus, cardiomyopathy, neuropathy, myopathy, diarrhea, vomiting (LS), lactic acidosis	Basal ganglia calcifications
**Coats plus syndrome** (*CTC1*) (Childhood/full/AR)	Developmental delay, epilepsy, spasticity, dystonia, ataxia	Retinal telangiectasia and exudates, osteopenia and fractures, gastrointestinal and hepatic bleeding, portal hypertension, bone marrow suppression Growth retardation	Calcification with leukoencephalopathy and brain cysts
**Aicardi-Goutières syndrome** (*ADAR*, *RNASEH2A*, *RNASEH2B*, *RNASEH2C*, *SAMHD1*, *TREX1*, *IFIH1*) (Childhood (usually after a short period of normal development)/full/AR/AD)	Dystonia, hypotonia, developmental delay Increased startle reaction	Acquired microcephaly, Hepatosplenomegaly Sterile pyrexias Chilblain lesions (ears, hands, fee) Skin mottling Increased liver enzyme, thrombocytopenia (transitory), interferon signature	Calcifications of basal ganglia and white matter Leukoencephalopathy Progressive cerebral atrophy
**Leukoencephalopathy, cystic, without megalencephaly** (*RNASET2*) (1 year/full/AR)	Seizure Neurological deterioration	Microcephaly	Brain calcification Cystic lesions (anterior, subcortical) Leukoencephalopathy
**Claudin-5** (*CLDN5*) (1 year/full/AR)	Seizure Developmental delay		Atrophy of the pons Calcification of the vertical pontine white matter bundles Cortical and basal ganglia calcification
**Hoyeraal Hreidarsson syndrome** (*DKC1*, *TINF2*) **and Congenital Dyskeratosis (Zinsser-Engman-Cole syndrome)** (*TERC*, *TERT*, *NHP2*, *NOP10*, *WRAP53*, *RTEL1*) (Childhood (first months)/full/AR X-linked/AD/AR)	Neurodevelopmental delay	Microcephaly Persistent pancytopenia Hepatic and pulmonary fibrosis Dyskeratosis (skin pigmentation, leukoplakia, toenail dystrophy) Scoliosis	Cerebellar hypoplasia Basal ganglia and subcortical calcifications
**3-hydroxyisobutyric aciduria** (1st decade/NA/NA)	Hypotonia and impaired neurological development	Microcephaly Facial dysmorphism Episodes of ketoacidosis	Migration brain disorder Calcification in the frontal and subependymal region and periventricular
**Inborn error of folates** **Metabolism** (*FOLR1*, *SLC46A1*, *MTHFR*, *DHFR*, *MTFD1*) (1st decade/full/AR)	Seizure Severe neurological deterioration	Leukopenia, megaloblastic bone marrow, hypoimmunoglobulinemia.	Demyelination Calcification of basal ganglia and white matter
**Carbonic anhydrase II deficiency syndrome** (*CA2*) (Congenital/full/AR)	Possible mental retardation	Osteopetrosis Renal tubular acidosis	Calcification of the basal ganglia, thalamus, subcortical white matter
**Collagen IV disorders** (*COL4A1*, *COL4A2*) (Childhood–adult/full/AD)	Seizure Ischemic or hemorrhagic events Migraine with aura (also isolated) Severe cases with intellectual disability and infantile motor symptoms Neuropathy, muscle cramps	Systemic vascular involvement (aneurysms) and symptoms (renal, ocular, muscular)	Microhemorrhage or and deep intracerebral hemorrhages Lacunar infarcts Dilated perivascular spaces Leukoencephalopathy Porencephaly Intracranial calcifications
**Cockayne syndrome** (*DDB2*, *ERCC1*, *ERCC2*, *ERCC3*, *ERCC4*, *ERCC5*, *ERCC6*, *ERCC8*, *GTF2H5*, *MPLKIP*, *POLH*, *XPA*, *XPC*) (1st decade/Full/AR)	Spectrum of conditions Neurodevelopmental and growth delay Impaired vision, hearing and severe neurological deficits	Cataract Scoliosis, contractures Arthrogryposis, microphthalmia	Calcification in the basal ganglia (possible)
**Nasu Hakola Disease—Polycystic lipomembranous osteodysplasia with sclerosing leukoencephalopathy 1 and 2** (*TYROBP*, *TREM2*) (3rd decade (bone changes) 4th decade (neurological symptoms)/full/AR)	Presenile dementia Parkinsonism Seizure	Cyst-like bone lesions	Brain atrophy Leukodystrophy Basal ganglia calcification
**Nakajo Nishimura syndrome** (*PSMB8*) (Childhood (Japanese)/full/AR)		Nodular-erythema like changes Lipodystrophy Finger Joint deformities Specific inflammatory signature and hyperpyrexia Possible cardiac alteration	Basal ganglia calcification Vessel calcifications (brain and systemic)
**Adams Oliver syndrome 2** (*DOCK6*) (Congenital/full/AR)	Epilepsy	Facial dysmorphism Ophthalmological abnormalities (congenital cataract, rod dystrophy, vitreoretinal abnormalities, optic atrophy) Aplasia cutis congenita of the scalp Terminal transverse limb defects Systemic abnormalities (cardiac, renal, hepatic)	Periventricular calcifications and ventricular enlargement
**Raine Syndrome** (*FAM20C*) **(77)** (Congenital/full/AR)	Neurodevelopmental delay Hydrocephalus Self-mutilating behavior	Microcephaly Facial dysmorphism Exophthalmos, gum hyperplasia, osteosclerosis	Punctiform periventricular and white matter calcifications
**Keutel Syndrome** (*MGP*) (Childhood/full/AR)	Developmental delay (mild) (epilepsy)	Dysmorphisms due to calcification of cartilages (facial, brachytelephalangism) Hearing loss Cardiovascular defects and pulmonary stenosis, respiratory symptoms	
**Fried Syndrome** (*AP1S2*) (Childhood/full/X-linked recessive)	Neurodevelopmental delay hydrocephalus Hypotonia, mental retardation, stereotypies	Microcephaly Short stature Facial dysmorphism Optic nerve atrophy	Basal ganglia calcification
***RAB39B*** (<40 years/full/X-linked)	Parkinsonism	Juvenile Parkinsonism Intellectual disability	Basal ganglia calcifications reported in some cases
**Hemorrhagic destruction of the brain, subependymal calcification, and cataracts** (*JAM3*)	Seizure Severe neurological deterioration Early death	Congenital cataract	Brain hemorrhagic destruction Subependymal calcification
**Pseudo-TORCH syndrome 1** (*OCLN*) (Congenital/full/AR)	Seizure Pyramidal signs Hypotonia	Microcephaly Possible dysmorphic features	Calcification of the white matter, basal ganglia, cerebellum, brain stem Cortical dysgenesis
**Pseudo-TORCH syndrome 2** (*USP18*) (Congenital/full/AR)	Seizure Neurological deterioration	Microcephaly Possible dysmorphic features Immune abnormalities Intravascular coagulopathy	Calcifications **Cerebral hemorrhage** Cortical dysgenesis
**Immunodeficiency 38 with basal ganglia calcification** (*ISG15*) (1st–2nd decade/full/AR)	Seizure	Recurrent infections	Basal ganglia and cortical calcification
**Spondyloenchondrodysplasia with immune dysregulation** (*ACP5*) (1st decade/full/AR)	Developmental delay Spasticity	Spondyloenchondrodysplasia Short statue Skeletal dysplasia Immune dysregulation (multiple infections and autoimmune diseases)	Late-onset cerebral calcification
**Rajab interstitial lung disease with brain calcifications** (*FARSA*, *FARSB*) (Infancy–early childhood/full/AR)	Delayed motor development Cognitive development can be preserved	Delayed growth Interstitial lung disease Systemic abnormalities (hepatic, renal, skeletal) Dysmorphic features (possible immune abnormalities)	Calcifications of the basal ganglia and cortex Possible leukoencephalopathy
**Seizures, early onset, with neurodegeneration and brain calcification** (*NRROS*) (1st decade/full/AR)	Seizure Developmental regression Pyramidal signs		Brain atrophy White matter abnormalities Calcifications Possible corpus callosum hypoplasia Possible cystic degeneration of the white matter
**Spastic paraplegia 56, autosomal recessive** (*CYP2U1*) (1st decade/full/AR)	Spastic paraplegia (and also upper limbs involvement) Dystonia Cognitive impairment	Macular dystrophy Pseudoxanthoma elasticum	White matter lesion Atrophy of the corpus callosum Calcifications of the globus pallidus
**Down syndrome** (Trisomy 21) (Congenital/full/IC)	Mental retardation Decreased muscle tone	Dysmorphic features, palm crease, large tongue, umbilical hernia, intestinal blockage, congenital heart disease	Basal ganglia (mostly globus pallidus)
**Pseudohypoparathyroidism** (*GNAS*, *PRKAR1A*, *PDE4D*, *PDE3A*) (1st–5th decade/full/AD)	Hypocalcemia tetani Seizure Mental retardation	Short stature Subcutaneous calcifications Brachydactyly Hypocalcemia, hyperphosphatemia, and increased serum concentration of PTH (PTH resistance) Normal renal function	Calcification of the basal ganglia, thalamus, subcortical white matter

**Table 2:** **Demographics and common comorbidities associated with brain calcifications syndrome.** The table shows the frequency of certain comorbidities that are found in brain calcifications syndrome that can help in the differential diagnosis. For specific references to each condition, please refer to the main text.

	Adult Onset	Pediatric Onset	Skin Abnormalities	Skeletal Abnormalities	Autoimmune/ Hematological Abnormalities	Pyrexia
Calcium metabolism disorder	✓	✓		✓		
SLE	✓				✓	
PFBC	✓	✓				
Aicardi- Goutières syndrome		✓	✓	✓	✓	✓
Coats plus syndrome		✓	✓	✓	✓	
Leukoencephalopathy, cystic, without megalencephaly		✓				
Claudin-5		✓				
Hoyeraal Hreidarsson syndrom		✓	✓	✓	✓	
Congenital Dyskeratosis (Zinsser-Engman-Cole syndrome)		✓	✓	✓	✓	
3-hydroxyisobutyric aciduria		✓				
Inborn error of folates Metabolism		✓			✓	
Carbonic anhydrase II deficiency syndrome		✓		✓		
Collagen IV disorders		✓				
Cockayne syndrome		✓		✓		
Polycystic lipomembranous osteodysplasia with sclerosing leukoencephalopathy 1 & 2 (Nasu Hakola Disease)		✓		✓		
Nakajo Nishimura syndrome x		✓	✓	✓	✓	
Adams Oliver syndrome 2		✓	✓		✓	
Raine Syndrome		✓		✓		
Keutel Syndrome		✓		✓		
Fried Syndrome		✓				
Hemorrhagic destruction of the brain, subependymal calcification, and cataracts		✓				
Pseudo-TORCH syndrome 1		✓				
Pseudo-TORCH syndrome 2		✓			✓	
Immunodeficiency 38 with basal ganglia calcification		✓			✓	
Spondyloenchondrodysplasia with immune dysregulation		✓		✓	✓	
Rajab interstitial lung disease with brain calcifications		✓				
Seizures, early-onset, with neurodegeneration and brain calcification		✓				
Spastic paraplegia 56, autosomal recessive		✓	✓			
Pseudohypoparathyroidism		✓				
Calcium metabolism disorder	✓	✓		✓		
SLE	✓				✓	
PFBC	✓	✓				
Aicardi- Goutières syndrome		✓	✓	✓	✓	✓
Coats plus syndrome		✓	✓	✓	✓	
Leukoencephalopathy, cystic, without megalencephaly		✓				
Claudin-5		✓				
Hoyeraal Hreidarsson syndrom		✓	✓	✓	✓	
Congenital Dyskeratosis (Zinsser-Engman-Cole syndrome)		✓	✓	✓	✓	
3-hydroxyisobutyric aciduria		✓				
Inborn error of folates Metabolism		✓			✓	
Carbonic anhydrase II deficiency syndrome		✓		✓		
Collagen IV disorders		✓				
Cockayne syndrome		✓		✓		
Polycystic lipomembranous osteodysplasia with sclerosing leukoencephalopathy 1 & 2 (Nasu Hakola Disease)		✓		✓		
Nakajo Nishimura syndrome x		✓	✓	✓	✓	
Adams Oliver syndrome 2		✓	✓		✓	
Raine Syndrome		✓		✓		
Keutel Syndrome		✓		✓		
Fried Syndrome		✓				
Hemorrhagic destruction of the brain, subependymal calcification, and cataracts		✓				
Pseudo-TORCH syndrome 1		✓				
Pseudo-TORCH syndrome 2		✓			✓	
Immunodeficiency 38 with basal ganglia calcification		✓			✓	
Spondyloenchondrodysplasia with immune dysregulation		✓		✓	✓	
Rajab interstitial lung disease with brain calcifications		✓				
Seizures, early-onset, with neurodegeneration and brain calcification		✓				
Spastic paraplegia 56, autosomal recessive		✓	✓			
Pseudohypoparathyroidism		✓				

## Data Availability

Not applicable.
